# Biology of an Adventive Population of the Armored Scale *Rhizaspidiotus donacis,* a Biological Control Agent of *Arundo donax* in California

**DOI:** 10.3390/insects12070588

**Published:** 2021-06-29

**Authors:** Charles A. Braman, Adam M. Lambert, A. Zeynep Özsoy, Ellen N. Hollstien, Kirsten A. Sheehy, Tara McKinnon, Patrick Moran, John F. Gaskin, John A. Goolsby, Thomas L. Dudley

**Affiliations:** 1 Marine Science Institute, University of California, Santa Barbara, CA 93106, USA; charliebraman@ucsb.edu (C.A.B.); ellen_hollstien@berkeley.edu (E.N.H.); kasheehy@ucdavis.edu (K.A.S.); taramckinnon@unr.edu (T.M.); tdudley@msi.ucsb.edu (T.L.D.); 2 Department of Biological Sciences, Colorado Mesa University, Grand Junction, CO 81501, USA; aozsoy@coloradomesa.edu; 3 Program of Hydrological Sciences, Department of Biology, University of Nevada, Reno, NV 89557, USA; 4 Invasive Species and Pollinator Health Research Unit, Agricultural Research Service, U.S. Department of Agriculture, Albany, CA 94710, USA; Patrick.moran@usda.gov; 5 Pest Management Research Unit, Agricultural Research Service, U.S. Department of Agriculture, Sidney, MT 59270, USA; john.gaskin@usda.gov; 6 Cattle Fever Tick Research Laboratory, Agricultural Research Service, U.S. Department of Agriculture, Edinburg, TX 78541, USA; john.goolsby@usda.gov

**Keywords:** *Arundo donax*, *Rhizaspidiotus donacis*, biological control of weeds

## Abstract

**Simple Summary:**

The invasive giant reed, *Arundo donax*, impacts river ecosystems world-wide. The plant-feeding scale insect *Rhizapidiotus donacis* is approved for biocontrol use in North America but a wild (adventive) population was found in southern California. We studied the adventive scale to document its distribution, life history, relatedness to European samples, risk to a native reed, and ability of a biocontrol wasp to develop within it. The adventive was found within a single watershed and is genetically closest to the Iberian scale population. *Rhizaspidiotus donacis* developed on some *Phragmites* reed types, but at lower densities than *Arundo*. It produces one generation each year with mobile juveniles from March through June. *Aphytis melinus* wasps showed similar interest in adventive *R. donacis* as their usual host with deposited eggs developing into a second generation. *Rhizaspidiotus donacis* appears limited in distribution by its life history, precluding broad biocontrol implementation through natural dispersal but allowing for targeted application. The genetic differentiation between imported biocontrol samples and adventive populations presents an opportunity for exploring benefits of hybrids and/or alternative genotypes where establishment has been difficult. While likely rare in the wild, spillover to vulnerable endemic *Phragmites* or deleterious parasitoid effects on scale populations warrants consideration when planning use of this agent.

**Abstract:**

*Arundo donax* (giant reed) is invasive in Mediterranean, sub-, and tropical riparian systems worldwide. The armored scale *Rhizaspidiotus donacis* is approved for biocontrol in North America, but an adventive population was recently discovered in southern California. We documented this population’s distribution, phylogeny, phenology, potential host spillover to *Phragmites spp*., and potential for parasitism by a common biocontrol parasitoid of citrus scale. The adventive scale was found within a single watershed and is genetically closest to Iberian scale genotypes. *Rhizaspidiotus donacis* developed on *Phragmites* haplotypes but at much lower densities than *Arundo*. The adventive population is univoltine, producing crawlers from March-June. *Aphytis melinus* parasitoids exhibited sustained interest in *R. donacis* during choice and no-choice trials and oviposition resulted in a small second generation. *Rhizaspidiotus donacis* appears limited in distribution by its univoltinism and sessile adult females. This presents challenges for broad biocontrol implementation but allows for targeted application. The genetic differentiation between imported biocontrol samples and adventive populations presents an opportunity for exploring benefits of hybrids and/or alternative genotypes where establishment has been difficult. While unlikely to occur in situ, spillover to vulnerable endemic *Phragmites* or deleterious parasitoid effects on scale biocontrol agents warrants consideration when planning use of *R. donacis*.

## 1. Introduction

*Arundo donax* L. (Poales: Poceae), giant reed, is a large-statured perennial grass originating in Eurasia [1] and now widely introduced into semi-arid riparian ecosystems throughout the world, including western North America from California to northern Mexico, Texas, and the southern U.S. [2,3]. It is targeted for control owing to numerous environmental and economic impacts including high evapotranspiration of limited water resources [4,5,6], promoting wildfire [7,8], and increasing channel erosion and sedimentation [9]. Further, *Ar. donax* causes displacement of native riparian vegetation and riparian-associated fauna [10], including sensitive wildlife species [11,12,13]. Conventional weed treatments have been implemented across its invasive range [14] but are expensive and entail extensive collateral damage in sensitive ecosystems [15]. Thus, a biological control program was initiated in the early 2000s to provide a more benign and cost-effective approach to its management [16,17,18].

Biocontrol development for *Ar. donax* initially focused on a stem-galling wasp, *Tetramesa romana* Walker (Hymenoptera: Eurytomidae), [17,18,19,20] and an armored scale insect, *Rhizaspidiotis donacis* Leonardi (Hemiptera: Diaspididae). Both are found on *Ar. donax* in the Mediterranean region where this grass is considered to be an ancient introduction [1,21]. Additional arthropod agents are under evaluation for biocontrol, including a leaf-mining fly, *Lasioptera donacis* Coutin (Diptera: Cecidomyiidae), which was released but did not establish [22], and a shoot-feeding chloropid fly, *Cryptonevra nigritarsis* Duda (Diptera: Chloropidae), which was rejected based on lab and field studies [23]. There have been preliminary investigations into the use of microbial pathogens for *Ar. donax* suppression [24]. Limited evidence suggests that a *Cryptonevra* species is present adventively in southern California [25]. Another insect herbivore, the aphid *Melanaphis donacis* (Passerini) (Hemiptera; Aphididae), is widespread in the invaded range of *Ar. donax* in California, as well as in Chile, and has modest impacts when present at high densities on leaf blades [25,26].

Early in the research program, *T. romana* was documented as being present adventively in southern California [25] and Texas [27,28], as well as in South Africa [29], so additional effort was directed to assaying the adventive insect material for comparison with those insects being imported and evaluating the relative benefits of expanding introduced ranges of both North American adventive populations by augmentative methods. In the lower Rio Grande Basin, the wasp has reduced live *Ar. donax* biomass by at least 32% since releases began in 2009, fostering partial recovery of native vegetation [19,30]. However, the impact of adventive *T. romana* in California was considered to be minor [31] due likely to reduced heat units for development compared to the lower Rio Grande Basin of Texas [32]. This gave credence to focusing attention on the arundo armored scale *Rhizaspidiotis donacis* (Hemiptera: Diaspididae).

At our long-term study area in southern California, *R. donacis* was documented in 2018 at a small number of locations, indicating another case of unintentional introduction of a specialist herbivore of *Ar. donax* prior to managed importation in this region. Thus, our research group sought to document the genetics, distribution, biology, and community interactions of this adventive form, in part to evaluate if it or the imported insects would perform better in this environment. A series of trials and experiments were designed to test the hypotheses that the adventive genotype did not differ from imported *R. donacis* in non-target host grass use, and that growth, phenology, and reproductive output did not differ substantially between the two types. Furthermore, given that the time and origin of the unintentional introduction are unknown, we conducted genetic assays to determine its geographic origin, genetic similarity to imported and archived *R. donacis* material, and whether genetic changes had taken place since its introduction.

Many *Ar. donax* stands are in fairly close proximity to commercial citrus groves, where a wasp, *Aphytis melinus* DeBach (Hymenoptera: Aphelinidae), has been widely released as a parasitoid of a citrus pest, the California red scale *Aonidiella aurantii* Maskell (Hemiptera: Diaspididae). This wasp is known to parasitize a variety of armored scales [33], so additional trials were conducted to determine if *R. donacis* may be within its fundamental host range. If so, non-target parasitism may play a role in both the rarity of the arundo armored scale and subsequently could constrain its potential for biocontrol of invasive *Ar. donax*.

We had 5 objectives: (1) document the known distribution of adventive *R. donacis* in riparian habitats throughout southern and central California; (2) compare the genetics of adventive, imported, and Mediterranean native range *R. donacis* to determine to what degree, if any, California populations are genetically distinct; (3) determine the phenology in situ of adventive *R. donacis* to determine how many generations occur annually and when juveniles potentially disperse; (4) confirm host fidelity of adventive *R. donacis* by trials using closely related native plant species; (5) determine the ability of *Ap. melinus* parasitoids to complete a developmental life cycle within adventive *R. donacis*.

## 2. Materials and Methods

### 2.1. Distribution

*Arundo donax* populations throughout coastal central and southern California were surveyed to assess presence and relative abundance of *R. donacis*. Locations were identified using a combination of the most recent aerial imagery hosted on Google Earth, and recorded observations on CalFlora (www.calflora.org) (accessed on 20 May 2018) and iNaturalist (www.inaturalist.org) (accessed on 20 May 2018). Sites from coastal San Luis Obispo to San Diego Counties were selected to extensively sample stands throughout this region, with higher intensity surveys occurring closer to the known adventive population in the Santa Clara River.

**Figure 1:**
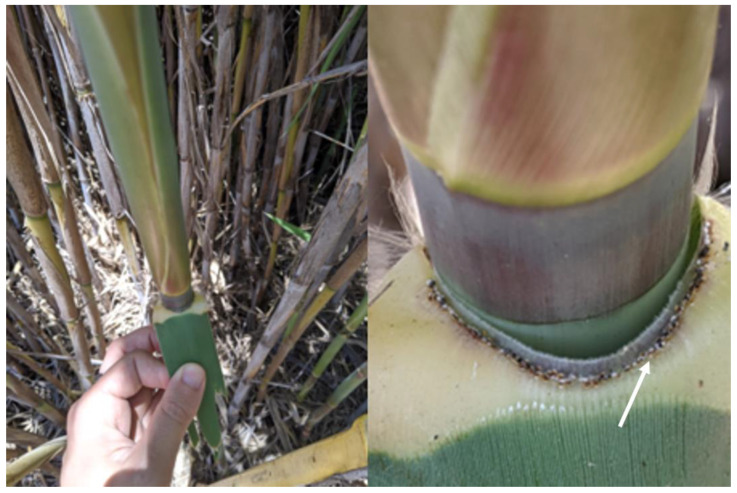
*Rhizaspidiotis donacis* attached along the leaf sheath of a mature *Arundo donax* stem. Scales (mainly immature males) can be found along the stem on leaf collars (indicated with arrow) up to one meter from the ground in the spring and summer. Both immature and mature females attach to the rhizome throughout the year.

The size and stem density of *Ar. donax* stands varied considerably among the 30 sites surveyed, and a minimum of 20 stems at sites with small populations (approximately less than 100 m^2^ in surface area) and 100 stems at sites with larger populations were inspected for scale presence. At each site, stems were haphazardly selected, but with an effort to inspect stems from across as much of the population as possible with half of visual evaluations made at the edge of the stand and half at least one meter in from the stand edge. Adult scales are readily visible throughout the year on rhizomes near the soil surface and as early 2nd instar male scales that often settle under the leaf sheaths near nodes along the length of the stem (Figure 1). Surveys occurred from June through September.

### 2.2. Rhizaspidiotis Donacis Genetics

**Figure 2:**
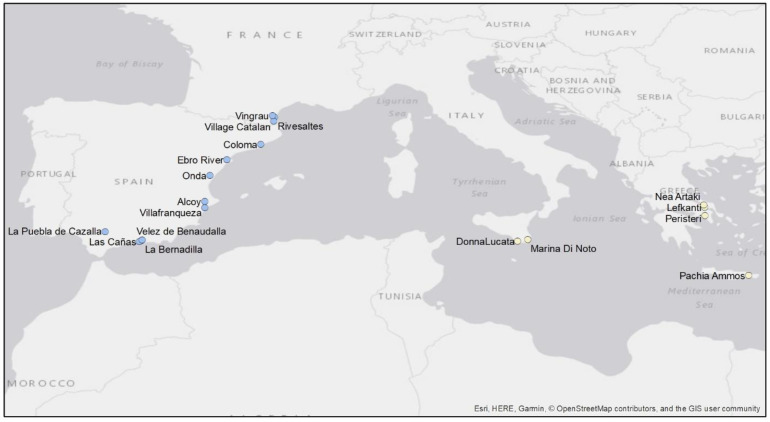
European sites where *Rhizaspidiotis donacis* specimens were collected between 2007 and 2010 for genetic analysis as part of the *Arundo donax* biological control program. Locations with the same color represent individuals that display less than 2% genetic distance.

Adult *R. donacis* specimens were collected in Europe between 2007 and 2010 as part of the *Ar. donax* biological control program [34,35]. The collection sites were Villafranqueza, Las Cañas, Alcoy, La Puebla de Cazalla, Velez de Benaudalla, La Bernadilla, Ebro River, Onda, Coloma in Spain; Vingrau, Village Catalan, and Rivesaltes in France; Donna Lucata and Marina di Noto in Italy; and, Lefkanti, Nea Artaki, Peristeri, and Pachia Ammos in Greece (Figure 2).

One individual scale insect from each of these locations was included in the analysis except for scale insects from Pachia Ammos, Greece, Rivesaltes, France and Donna Lucata, Ital where two specimens were analyzed. Twenty adults of the adventive scale population in Santa Paula, CA, USA were collected from rhizomes for genetic comparison to the European samples described above. Of these, seven individuals each collected from separate plants had their DNA successfully isolated, amplified and sequenced. We also included in the analysis one specimen of one of the scale haplotypes (Villafranqueza, Spain) used for releases in Texas and northern California as part of the USDA biological control program. The analyzed Villafranqueza, Spain females were 3rd or 4th generation progeny of crawlers collected from females that had been collected and shipped from Spain in early 2018 into the USDA-ARS Invasive Species and Pollinator Health Research Unit Arthropods Quarantine Laboratory in Albany, CA. The crawlers (approximately 200 per plant) had emerged from females isolated from rhizomes in gelatin capsules in quarantine and had then been released onto small *Ar. donax* plants [36] in a non-quarantine greenhouse at the USDA-ARS in Albany, CA. Potted plants with mature females were transported to the University of California, Santa Barbara Greenhouse for further propagation in September 2018.

All scale specimens were stored in 95% ethanol prior to analysis and scale DNA was isolated using DNeasy Blood and Tissue Kit (Qiagen Corporation, Valencia, CA, USA) following their protocol except for the two consecutive 40 µL elutions in Tris-acetate-EDTA buffer to ensure higher DNA concentration.

The CO1/CO2 region was amplified using the primer pair c1-J-2753ywr [37] and c2-N-3662 [38]. The polymerase chain reactions contained NEB Taq polymerase (1.25 units), 1× ThermoPol Buffer (New England Biolabs, Ipswich, MA, USA), 0.2 µM of each primer, 0.2 mM dNTPs, 1 µL of genomic DNA and water to 30 µL. Initial denaturation was at 94 °C for five minutes followed by 40 cycles of denaturation (95 °C for 45 s), annealing (52 °C for 60 s) and extension (72 °C for 60 s). The final extension was five minutes at 72 °C. The amplified DNA was run on a 1% agarose gel (1× TAE) and gel extracted using a QIAquick Gel Extraction Kit (Qiagen Corp, Valencia, CA, USA). The resulting DNA fragments were sequenced by Genewiz, Inc. (South Plainfield, NJ, USA) using the same primers as the PCR amplification. Sequencher^®^ (version 5.0.1 DNA sequence software, Gene Codes Corporation, Ann Arbor, MI, USA) was used to assemble the CO1/CO2 regions.

The obtained DNA sequences of the scale insects collected in Europe and California are registered in GenBank database under the accession numbers MW854384-MW854405. Maximum Parsimony analysis of the resulting dataset was performed using PAUP* v. 4.0a [39]. The heuristic Maximum Parsimony (MP) search employed 500 random taxon addition sequences and the tree-bisection-reconnection branch-swapping algorithm. All characters were weighed equally and there were no insertion/deletion events. A 5000-replicate fast stepwise-addition bootstrap analysis was conducted to assess clade support. *Aspidiotus nerii* Bouché (Hemiptera: Diaspididae) (GenBank accession number GU213902.1) was included in this analysis as the outgroup.

Population Analysis with Reticulate Trees (PopART, http://popart.otago.ac.nz) (accessed on 7 October 2020) was used to construct a haplotype network with the TCS option to evaluate the genetic distribution of the different haplotypes. The localities of specimens were added manually. Estimates of evolutionary divergence between sequences were calculated as the minimum uncorrected p-distances using MEGA X: Molecular Evolutionary Genetics Analysis [40] across computing platforms.

### 2.3. Adventive Rhizaspidiotis Donacis Phenology

**Figure 3:**
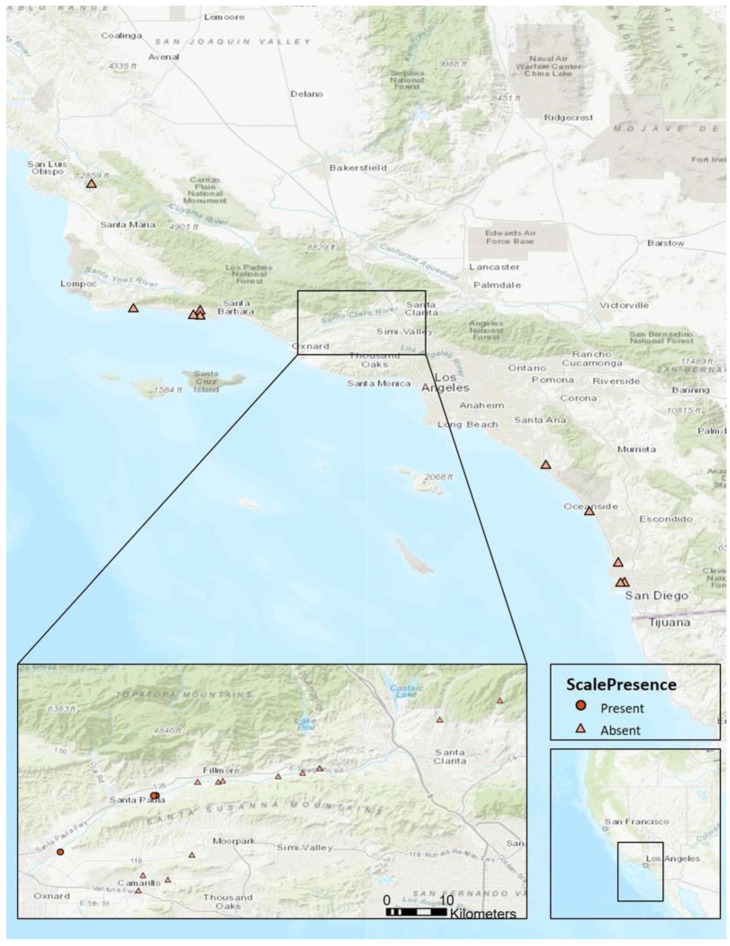
Distribution of *Rhizaspidiotus donacis* throughout central and southern California. Surveys were conducted in 2018–2020. Trials conducted with adventive scales were performed with individuals collected from the population near Santa Paula.

The timing of life stages of the only known adventive *R. donacis* population was evaluated on *Ar. donax* rhizomes collected near the Santa Clara River, Santa Paula, California (Figure 3). From March 2019 through July 2020, rhizomes were excavated from the soil every two weeks and stored at 4 °C until processing. Scale developmental stages were assessed approximately weekly (average interval = 10.42 days). Fifty live scales were haphazardly sampled on the collected rhizomes as encountered and dissected to determine life stage and sex, with categories based on life stage descriptions from Moran and Goolsby [34]. The presence of crawlers was also noted.

### 2.4. Rhizaspidiotis Donacis Host Range

The adventive scale’s ability to establish on related or morphologically similar host plants was tested in a no-choice controlled greenhouse study at the University of California, Santa Barbara. Forty-four plants constituting eleven *Phragmites australis* (Cav.) haplotypes were evaluated, including specimens of the native, introduced, and Gulf Coast lineages *sensu* Saltonstall [41], as well as a native X introduced hybrid from Las Vegas, NV [42]. Plants were selected from haplotypes growing at our greenhouse at the University of California, Santa Barbara. This collection was sourced from throughout the United States with a focus on western populations [43] and all individuals have been maintained at the facility for at least three years. Prior to the study, all plants were cut back to the soil surface to promote new stem growth with plants grouped into categories of native North American lineage *P. australis* ssp. *americanus* (22 plants), introduced European lineage (haplotype M, 11 plants), and the native x introduced hybrid from Las Vegas, Nevada (5 plants). Two plants each of *Ar. donax* and the horticultural perennial grass *Arundo formosana* Hack were also tested. Plants were started from rhizomes in a 3:1 soil:sand mix and kept in a greenhouse (20.9 °C average temperature, 45% RH) throughout the experiment on a timed drip line irrigation system with supplemental spray watering as needed.

Release of adventive *R. donacis* onto each plant was performed with a modified protocol of Goolsby et al. [44]. For each plant, two introductions of the mobile crawler phase of *R. donacis* occurred for each potted plant. Crawlers were obtained from mature female scales on *Ar. donax* rhizomes collected from along the Santa Clara River as described above. Between 10 and 20 live mature mother scales with visible signs of crawlers were placed into individual gel capsules. The first release occurred on 3 May 2019 by pinning two opened capsules to each plant near the base of a live stem. Fifteen days after the initial release, an additional four opened gel capsules with scales from a second collection of rhizomes were also attached to each plant. Eleven weeks after the initial introduction, plants were evaluated for scale establishment and development. Above-ground biomass of test plants was removed and final scale counts and developmental stages were assessed after 19 weeks (17–27 September 2019). We visually assessed scale densities for week 11 count, using a categorical scale (0–10 low, 11–30 medium, >30 high) to avoid handling plants and possibly dislodging scale.

We conducted a parallel test using Rivesaltes, France scale in USDA greenhouses in Albany, CA, in March 2020 following Goolsby et al. [44]. Potted plants of *P. australis*, including native haplotype H (Santa Paula, CA, USA) or B (Little Caliente Springs, CA, USA), Gulf Coast haplotype I (Salton Sea, CA, USA), or European haplotype M or the native X introduced hybrid (Las Vegas, NV, USA) were established using rhizomes provided from the University of California, Santa Barbara collection described above. Rhizomes were planted in a 3:1 soil:sand mixture and were maintained in the greenhouse (19.8 °C average temperature, 60% RH) using drip irrigation for four weeks before infestation. Crawlers collected from Rivesaltes, France females were collected into gelcaps and placed on the bases of stems by pinning gelcaps to the stems. Between 200–233 (avg 214 ± 1.4) crawlers were placed on each plant. Above-ground plant parts were dissected and final counts of 3rd instar (adult) females made after six months. Progeny adult females were isolated in groups of up to 15 in gelatin capsules and held in a growth chamber at 25 °C for 8 weeks. Crawlers were counted and removed every three to five days.

### 2.5. Aphytis Melinus Parasitism

Large numbers of the parasitoid wasp *Ap. melinus* are released annually around the Santa Clara River basin (Brett Chandler, personal communication), and broadly across California for control of pest citrus scale species. We evaluated the potential for *Ap. melinus* to use *R. donacis* as a host relative to another locally common scale insect in citrus, the California red scale *Ao. aurantii* (a preferred host of *Ap. melinus* collected from citrus trees in Goleta, CA, USA). Two sets of lab trials were conducted to determine if *Ap. melinus* would interact with and could develop on *R. donacis.*

In the first set of trials, we compared *Ap. melinus* host-use behavior when exposed to each scale species in paired choice and no-choice chambers. Chambers were constructed using glass slides (75.0 mm by 51.0 mm) glued together leaving a single 17 mm wide opening at one side sealed with parafilm after organism introduction. In choice trials, chambers had three *R. donacis* and three *Ao. aurantii* scales with the anterior of the body glued to the slide with honey, which also acted as a food resource for wasps [45]. No-choice trial chambers had either six *R. donacis* or six *Ao. aurantii* scales. Approximately 20 *Ap. melinus* adults were introduced to each chamber. Behavior was observed for 30 min intervals at introduction and after 60 and 120 min with counts combined for analysis. Behaviors were noted as either inspecting or probing. Probing only occurs post-inspection and is typically followed by oviposition behavior, however, organisms could not be dissected during trials to check for eggs so probing was the highest behavior noted. Inspection was designated as any event in which *Ap. melinus* antennated a scale or demonstrated a turning pattern while walking on the scale. Probing was designated as any instance where *Ap. melinus* vertically positioned its ovipositor while touching the scale followed by dorso-ventral vibrations as described by Luck et al. [46]. Behaviors are sequential, so the last behavior was noted at each incident. Eight choice trial replicates were conducted. Twenty-two and thirteen no-choice replicates were conducted for *R. donacis* and *Ao. aurantii*, respectively.

In the second set of trials using only *R. donacis,* we closely observed *Ap. melinus* behavior with continued daily monitoring of chambers after the death of *Ap. melinus* introduced into each chamber to determine if a second generation would successfully develop. The anterior surfaces of 10 *R. donacis* were adhered to a Petri dish with honey and 20 *Ap. melinus* introduced into each dish to observe inspection and probing behaviors over a three-day period. Observations began at introduction, with each observation event occurring for ten minutes each hour between 0900 and 1600 as possible, with 23 observations per dish. A total of three replicate dishes were observed. Behaviors were recorded as in Trial 1, as well as events where *Ap. melinus* walked onto, but did not antennate or probe scales.

Inspection and probing data were not normally distributed (Shapiro-Wilk test), so a non-parametric Kruskal-Wallis test was used to evaluate differences in *Ap. melinus* behavior between host species. Analyses were performed in R version 4.0.3 [47].

## 3. Results

### 3.1. Distribution

Adventive *R. donacis* were documented at three of the sites surveyed and appear to be restricted to the Ventura County reach of the Santa Clara River (Table 1, Figure 3). Two of the locations (Balcom and Saticoy) were on dry river terraces with sandy soil. Plants at these locations were drought stressed and had stunted stems and few leaves. The third location (Taylor Property) was approximately 350 m from the other Santa Paula site (Balcom) and had large, robust stands with perennially moist soils and higher silt and organic content. There were no other detections of this scale in focused surveys in coastal, inland, and desert regions of California.

### 3.2. Rhizaspidiotis Donacis Genetics

**Figure 4:**
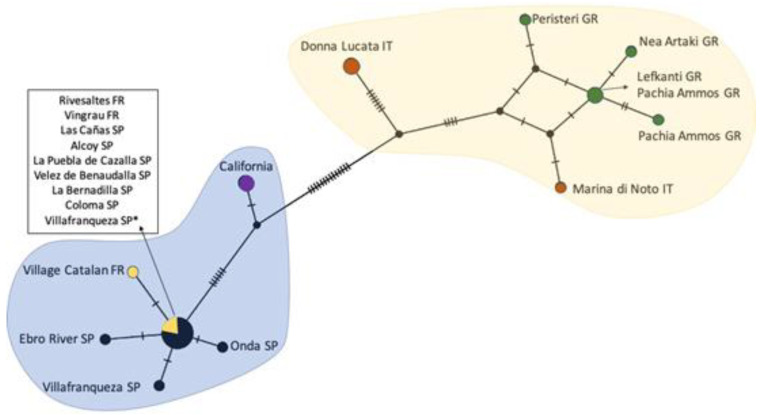
Haplotype network of *Rhizaspidiotus donacis* populations collected from California, France (FR), Greece (GR), Italy (IT), and Spain (SP) plotted with PopART and localities added manually. A 782 base pair sequence from scales collected at each location was compared via haplotype network analysis. Locations are indicated by the name of the collection location site. Villafranqueza SP* is the specimen from the population collected for release as part of the biological control program. Each hatch mark indicates a single nucleotide change. The colored shapes encompass sequences that display less than 2% genetic distance in Table S1. Each country where samples were collected is indicated by a unique color.

**Figure 5:**
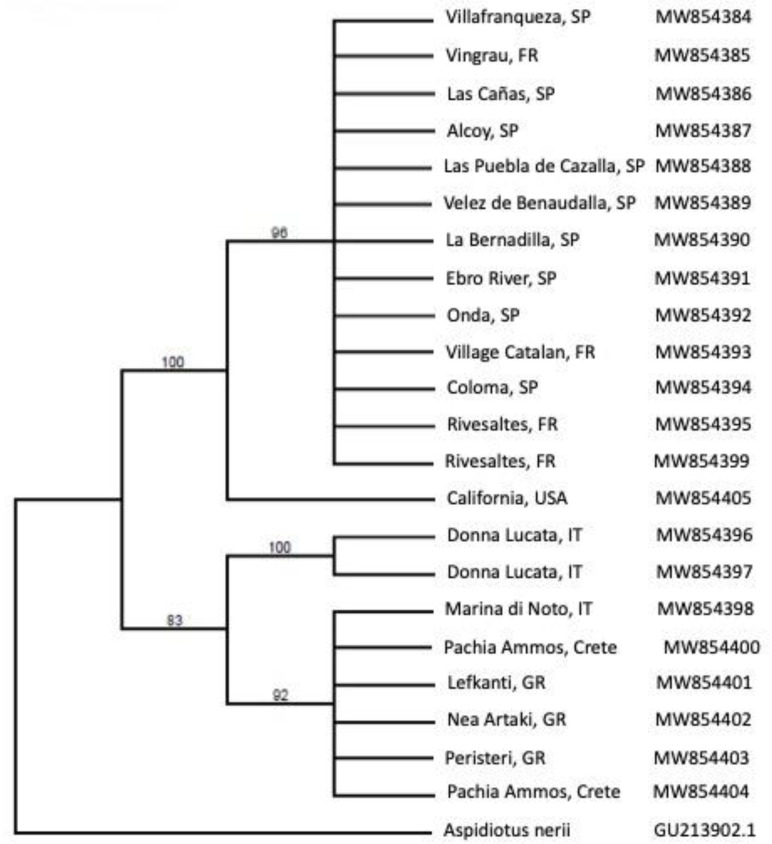
Maximum parsimony 50% majority consensus cladogram of 127 trees of cytochrome c oxidase subunits 1 and 2 mtDNA region for *Rhizaspidiotus donacis* and outgroup (*Aspidiotus nerii*). Bootstrap values are shown above branches. Collection sites and GenBank accession numbers are at the tips of the tree.

A total of 42 *R. donacis* mitochondrial CO1/CO2 regions were analyzed, comparing 782 bases to the same regions of reference material from two main native regions located in Spain/France and Italy/Greece. All of the 20 scales collected and sequenced from the adventive California population were identical along the entire 782 base pair fragments, indicating a likely clonal population. One specimen originally collected in Villafranqueza, Spain for releases in Texas and California as part of the USDA biological control program (Figure 4, designated with an asterisk) had one nucleotide difference compared to another specimen collected in the same location suggesting possible genetic variation in the area. Both specimens are included in the haplotype network (Figure 4). Maximum parsimony analysis covered 782 bases, of which 34 base positions were parsimony-informative (Figure 4 and Figure 5).

The haplotype network (Figure 4) indicates the adventive California population is more closely related to the samples collected from the Spain/France region, but are genetically distinct, suggesting that they are either from a different source location or may be developing into a distinct genotype or lineage. The uncorrected *p* genetic distance analysis showed less than 2% difference between the adventive *R. donacis* and the Spain/France haplotype, whereas the genetic distance was higher than 2% compared to the Italy/Greece haplotype.

### 3.3. Adventive Scale Phenology

**Figure 6:**
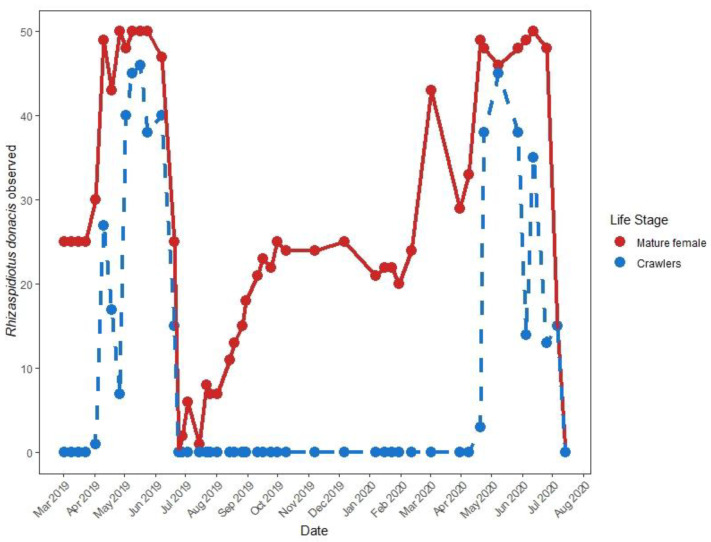
Adventive southern California *Rhizaspidiotus donacis* adult and crawler seasonality of life stages. Each sample point shows how many of 50 encountered *R. donacis* were mature females (red solid line) and how many females had crawlers present internally or within the scale test (blue dashed line).

The adventive population displayed a univoltine life cycle with the mobile crawlers present from April to July (Figure 6), coinciding with the peak abundance of mature adult females. In the spring after crawler emergence, males could be routinely observed on the nodes and ligules along the length of stems.

### 3.4. Host Range Trials

In host range tests in greenhouses at the University of California, Santa Barbara all *Ar. donax* test plants had high densities of early instar scales at the initial monitoring date in July and adult females present at the September end date (Table 2). Of the *Phragmites* lineages, most plants from the *P. australis* subsp. *americanus* lineage (18 of 22) had scale on them after 11 weeks. By week 19, ten plants had mature adult scales, with plants from each haplotype tested still infested. Asian and Gulf Coast *P. australis* had scales present at first check but no scale survived to maturity. Introduced *Phragmites* had low to high densities of scale on 9 of 11 plants at first check, with one plant hosting adult scales at final check. Hybrid *Phragmites* had low to medium densities of early instar scales on four of five test plants in July, with three plants hosting mature adult females at the September date. *Arundo formosana* had no scale establishment at either observation date. In the greenhouse trial at the USDA Albany, CA facility, two of the six plants of *P. australis* subsp. *americanus* (native) supported development of six total adult females, and four females isolated from one of those plants produced 10 crawlers (Table 3). No adult females developed on any of three other subspecies/haplotypes of *P. australis*. All eight *Ar. donax* plants tested supported development of adult females after 6 months, at a density 27-fold higher than that on the one native *P. australis* subsp. *americanus* accession that supported adult females (Table 2). Females from five of eight *Ar. donax* produced over 1000 crawlers, a density per plant 74-fold higher than one of four *P. australis* subspecies/haplotypes that produced crawlers (Table 3).

### 3.5. Aphytis Melinus Parasitism

In trials where *Ap. melinus* was given a choice between *Ao. aurantii* and *R. donacis*, there was no difference in inspection (Kruskal-Wallis, χ = 0.501, df = 1, *p* = 0.48) or probing rates (Kruskal-Wallis, χ = 0.235, df = 1, *p* = 0.63) between the two host species. In no-choice trials, there was no difference between inspection (Kruskal-Wallis, χ = 0.023, df = 1, *p* = 0.88) or probing (Kruskal-Wallis, χ = 0.026, df = 1, *p* = 0.87) rates between host species; however, inspection events were more common across both scale species than probing attempts. The inspection events were similar for both *R. donacis* (mean = 0.909 per hour) and *Ao. aurantii* (1.0 per hour). The rate of probing events was also comparable for *R. donacis* (mean = 0.36 per hour) and *Ao. aurantii* (0.54 per hour).

**Figure 7:**
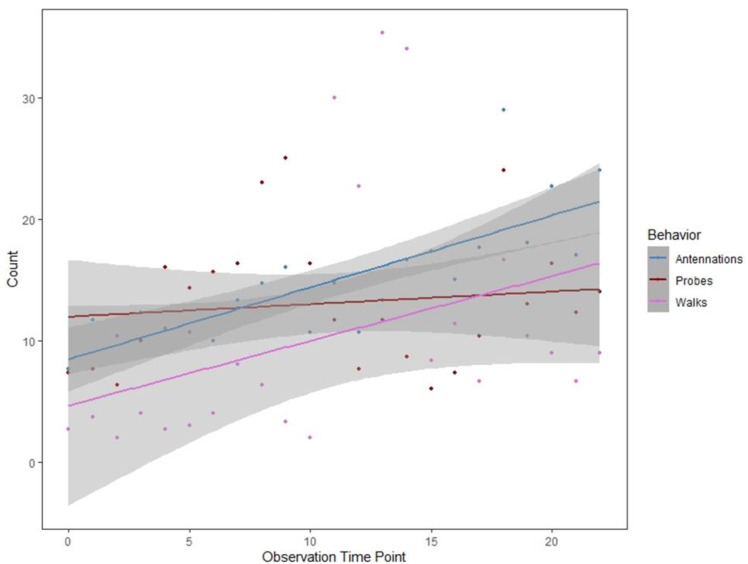
Regression of *Aphytis melinus* inspection and probing behavior on *Rhizaspidiotus donacis* hosts over a 3-day period. Shaded areas represent 95% confidence intervals. Parasitoids showed both immediate and sustained interest in hosts, with all three chambers producing a second generation of adults.

When observed for multiple days in no-choice chambers, *Ap. melinus* showed sustained walking-over, antennation, and probing behaviors with the frequency of each behavior increasing over time (Figure 7). Twenty days after introduction, all of the original *Ap. melinus* were dead, but all three arena dishes had live second-generation adults.

## 4. Discussion

### 4.1. Distribution

*Rhizaspidious donacis* was only identified as being adventive in North America in 2018, and this study was intended as an initial evaluation of its biology for comparison with material that USDA-ARS has been testing for several years in Texas and California [49,50,51]. Scale imported and released in Texas and California have limited mobility, under 10m within the first five years of release [50] and this appears also to be the case for the adventive genotype. In greenhouse studies, it was clear that dispersal abilities of the fragile, short-lived (~2 days, [50]) crawler stage would be limited, and adult females are sessile. The short-lived male alate has greater dispersal capacity but does not disperse the species if reproductive females are absent at touchdown sites (Trials to document the dispersal capacity of the scales present at our Santa Clara River field sites were suspended due to the Covid-19 pandemic). Limited dispersal capability is a likely factor explaining the very restricted distribution of the adventive scales, which have only been found near the city of Santa Paula and downstream at the Santa Clara River in southern California despite extensive searches in California (Figure 3). Dispersal of the adventive population is likely dependent on the limited mobility of crawlers and stochastic river scouring events that could transport infested *Ar. donax* rhizomes and stems downstream to new suitable sites but would only cause dispersal if gravid adult females are present on the debris and they survive the journey. Either type of distribution event to other rivers seems unlikely given substantial distances between river catchments suitable for *Ar. donax* infestation and the difficulty of dispersing more than a few meters by individual scale insects. In its native range, *R. donacis* is more abundant at sites with stable plant populations and mostly dry soil than at sites with perennially wet soil [52]. In this study, adventive scales were found in two dry and one moister site(s).

If the management objective is widespread establishment of *R. donacis* for control of widely dispersed *Ar. donax*, then its low dispersal capability is a barrier to landscape-level control which would require labor-intensive re-distribution measures [53]. Conversely, limited dispersal capacity could be a desirable trait in some IPM applications, not just because of the relatively low probability of encountering non-target hosts, but also because this could improve spatial targeting of releases.

### 4.2. Genetics

Genetic analysis suggests that the adventive *R. donacis* most likely originated from the western Mediterranean region. *Arundo donax* was intentionally introduced into North America by the early 1800s [54]. If this adventive scale population was also introduced at this time, it could have experienced a large number (perhaps >100) of reproductive cohorts, during which time local adaptation could potentially generate genotypic and corresponding phenotypic differences from the originating populations. That suggests the adventive genotype may be better adapted to climate conditions in this region and could be the subject for re-distribution to other *Ar. donax*-infested sites. However, we do not know the specific introduction date, and commercial growing operations introduced new *Ar. donax* clones through the 1900s (S. Hedrick, personal communication).

The biocontrol program may benefit from genetic mixing of the two *R. donacis* genotypes to potentially yield a degree of hybrid vigor [55,56]. There is early but not conclusive evidence of establishment of imported *R. donacis* haplotypes from Rivesaltes, France and Villafranqueza, Spain at seven sites in northern California (P. Moran, unpubl. data). The imported material comes from a limited area of southern Europe, while the highly localized range and population size of the adventive scale similarly limits the population’s gene diversity that could facilitate further adaptation in the southwestern U.S. There is no overlap currently in distributions of these genotypes such that field hybridization occurs, but hybridization could be applied under lab conditions. However, the univoltine life cycle means that selection for desirable traits would be a relatively slow process.

### 4.3. Phenology

The adventive insect in California was shown to be univoltine (Figure 6). Our results align with phenology assessments of Iberian *R. donacis* populations, where scales exhibited one generation per year with crawlers present from April through July [57]. This contrasts to the more rapid development of *R. donacis* imported from Europe for *Ar. donax* control, which under greenhouse conditions in Texas produced two generations in a year, reproducing in March and November [34,36]. However, voltinism of established field populations in Texas has not been systematically assessed. Development rates of Diaspididae are strongly influenced by thermal regime [58] and modest differences in environmental conditions often can determine whether an insect is univoltine or bivoltine [59]. The moderate difference in thermal regimes between sites in Texas (warmer) and California (cooler) is considered to be a primary reason for greater population growth of *T. romana*, the other biocontrol agent targeted at *Ar. donax* in Texas [32]. Tests with adventive and introduced scale under similar controlled conditions are needed to confirm growth and reproductive differences. However, there could also be subtle differences in adult female development rate or crawler yield depending on the source population of *Ar. donax* used [52].

### 4.4. Host Range

Adventive *R. donacis* in California feeds primarily on *Ar. donax* but could be considered weakly monophagous based on our greenhouse host choice trials (Table 2). The biocontrol strain showed some utilization of non-target grass species, including the introduced *P. australis* lineage (haplotype M) in North America, but adult female scale insect abundances were five to eight-fold lower on this non-target plant, and the level of risk was deemed sufficient for regulatory approval for field release [44]. In our study, under greenhouse conditions, the adventive form of *R. donacis*, as well as the French accession used in field releases as a biocontrol agent in Texas and California, were able to develop to maturity in no-choice trials on some, but not all, varieties of *P. australis* including both native and non-native haplotypes. Development of the French accession to the adult female stage was 27-fold lower on the native *P. australis* H haplotype than on *Ar. donax*, and production of crawlers was 74-fold lower. Similarly, Goolsby et al. [44] found that development to the adult female stage was substantially lower on non-*Ar. donax* hosts, with about a 10-fold decrease on the non-native *P. australis* M haplotype and 20-fold decrease on native haplotypes.

The low, but nonzero, host use of *P. australis* subsp. *americanus* is significant because this subspecies is a taxon of conservation concern in North America [43,60]. Efforts to implement biocontrol for the introduced *P. australis* haplotype are delayed owing to concerns over potential unintended impacts to native common reed populations [61], particularly in western North America where the native lineage is increasingly rare and threatened by hybridization [42,43]. In studies at two field sites along the Rio Grande in Texas, the scale is well-established on *Ar. donax*, yet no *R. donacis* was found on any of the 112 *P. australis* ssp. *berlandieri* (Gulf Coast haplotype I) plants located between 0 and 10 m from the closest infested *Ar. donax* [51]. Similar surveys could be conducted in California, but are limited by the localized distribution of *R. donacis* and low abundance of *P. australis* near these scale populations. Alternatively, field host use could be evaluated on *P. australis* planted adjacent to *Ar. donax* infested with armored scale. Differences in predicted and realized field host range occur in weed biological control agents; of 493 agents released worldwide through 2016, about 12% were found in the field on non-target plants, but only 25% of these cases involved sustained population presence on the nontarget, and fewer than 1% caused damage sufficient to alter nontarget plant life history [62,63]. Given the need for *R. donacis* to build dense populations in release plots to exert impact on *Ar. donax* [50], the probability of this armored scale negatively impacting *P. australis* in the U.S. is low.

Nonetheless, we urge caution in implementing biocontrol of *Ar. donax* in the western region of the U.S. using *R. donacis*, particularly the adventive form, owing to the potential for limited, but potentially significant, non-target effects to related large-statured grasses. Future field choice trials may partially assuage this concern, particularly for sites where *Ar. donax* may be in close proximity to native common reeds.

### 4.5. Aphytis Parasitoids

Another possible explanation for the limited distribution of adventive scales is population regulation by parasitoids already present in the infested range. Many parasitoids of armored scales tend to be moderately polyphagous in host use, including *Ap. melinus* introduced in much of California for biological control of red scale (*Ao. aurantii*) on citrus [33]. Using *Ap. melinus* commercially produced for field releases, thereby avoiding potential confusion if cryptic species were present in field-collected populations [64], this parasitoid displayed similar pre-oviposition behavior toward both *R. donacis* and the ‘preferred’ host, *Ao. aurantii* tested as a control in both choice and no-choice trials. Wasps exhibited immediate and sustained interest in *R. donacis* as a host, and in lab trials *R. donacis* supported development of *Ap. melinus* to maturity.

The distribution of *Ap. melinus* is presumably patchy across the range of *Ar. donax*, with overlap most likely in riparian areas with nearby citrus production. It is also unclear how widely *Ap. melinus* can disperse from release points or if they have naturalized populations in these areas. While these interactions in situ among California’s riparian areas are likely less frequent and intense than in our lab trials, they point to the possibility of diminished efficacy of both forms of *R. donacis* as biocontrol agents used in close proximity to *Ap. melinus*. Further, current *Ap. melinus* use in the Santa Clara River valley may be contributing to the limited establishment and expansion of adventive *R. donacis*.

### 4.6. Synthesis

The biological control program for *Ar. donax* has focused on *T. romana, R. donacis*, and *L. donacis* [17,22]. An objective of considering multiple agents, besides ‘bet-hedging’ on at least one agent proving effective, is to introduce specialist herbivores that feed on different plant parts or fill different niches, thereby synergistically enhancing host biocontrol [65,66,67]. Agent selection with respect to climate diversity in the introduced range is also important, as in the case of the armored scale and the leafminer *L. donacis*, which may adapt more readily in cooler, more temperate *Ar. donax*-invaded areas. Similarly, *T. romana* has developed large populations with impact [19,30] in the Lower Rio Grande Basin of Texas and Mexico, in part due to heat units that are higher there than in the native range or in California [32].

The potential for re-distributing an herbivore that is already present in the invasive range reduces the regulatory burden for biocontrol implementation, so the adventive scale offers an opportunity to apply what is, effectively, a form of augmentative biocontrol [68] to enhance *R. donacis* population size, range, and efficacy (e.g., [69]). The ecological constraints on agent establishment should be lower for herbivores already adapted to local environmental conditions. Though more often applied to management of pest arthropods [70], augmentation offers a valuable option for suppressing alien weeds. This does not preclude the subsequent importation of genetic variants of the same herbivore taxa, particularly if there are management benefits of access to multiple agent haplotypes [71].

There remain advantages (and disadvantages) to the use of multiple genotypes and multiple species for biological control of weeds [72], particularly for weeds distributed across wide geographic ranges and environmental gradients [73]; c.f. [74]. Continued development of available genetic forms of *R. donacis*, as well as other agents, is well-advised, with the caveat that with those herbivores already present and apparently not causing substantial weed damage, it would be prudent to consider other taxa anticipated to have greater impact.

## 5. Conclusions

An adventive form of the arundo armored scale, *R. donacis*, found in at least one southern California watershed, is sufficiently similar in life history and host choice to be considered as an alternative form of this specialist herbivore for the biological control of invasive *Ar. donax*. This adventive form may be useful for biocontrol management at *Ar. donax*-infested California wetland sites where the approved, imported biocontrol conspecific agent is inaccessible and/or fails to establish. As a genetic form of the species already adapted to the southern California environment, it may better tolerate local conditions than the European material. Intentional hybridization of the Californian and European forms could be done to enhance genetic diversity and thereby accelerate the capacity of this agent to adapt to novel conditions but has not yet been attempted. Further investigation should, however, consider potential non-target use of native *P. australis*, and possible *R. donacis* population regulation imposed by extant natural enemies, including the generalist parasitoid, *A. melinus*, present across the target region as a biocontrol agent against scale insects on citrus.

## Figures and Tables

**Table 1:** Locations and coordinates (WGS 1984) of *Arundo donax* stands sampled for *Rhizaspidiotus donacis* throughout central and southern California. *Rhizaspidiotus donacis* was found only at locations with bold text.

Location	City	Latitude	Longitude
Little Falls Creek	San Luis Obispo	35.24578	−120.48773
Bouquet Canyon	Valencia	34.51580	−118.44873
Whispering Pines	Valencia	34.48612	−118.54285
Refugio Creek	Goleta	34.47301	−120.06892
Gaviota Beach	Gaviota	34.47134	−120.22722
San Jose Creek	Santa Barbara	34.45812	−119.80940
Santa Barbara Airport	Santa Barbara	34.42988	−119.85166
Lower Maria Ygnacio Creek	Santa Barbara	34.42648	−119.80915
Atascadero	Santa Barbara	34.42525	−119.81018
Santa Clara River, Route 126	Piru	34.41005	−118.73131
Newhall Orchards	Newhall	34.41000	−118.73174
Rancho Camulos	Piru	34.40282	−118.75805
Santa Clara River	Piru	34.39702	−118.79628
Sespe Cienega Site 2	Fillmore	34.39008	−118.88324
School Farm	Fillmore	34.38875	−118.92273
Sespe Cienega	Fillmore	34.38854	−118.89019
**Balcom**	**Santa Paula**	**34.36631**	**−118.98730**
**Taylor property**	**Santa Paula**	**34.36560**	**−118.99095**
**Saticoy**	**Ventura**	**34.27727**	**−119.13735**
Calleguas Creek 3	Somis	34.27365	−118.93174
Calleguas Creek 2	Somis	34.24199	−119.00829
Arroyo Conejo	Camarillo	34.23498	−118.96936
Calleguas Creek	Camarillo	34.21831	−119.01564
San Juan Creek	San Juan Capistrano	33.49504	−117.65840
Oceanside	Oceanside	33.20625	−117.38623
Carroll Canyon	San Diego	32.88784	−117.20662
Mission Valley	San Diego	32.76488	−117.16893
Friars Road	San Diego	32.76246	−117.19450

**Table 2:** Host range trials of adventive *Rhizaspidiotis donacis* focusing on haplotypes of *Phragmites australis*, the closest relative of *Arundo donax* in North American, and two horticultural grass species. Trials were conducted in a greenhouse at University of California, Santa Barbara. Plants were checked for establishment at 11 weeks and 19 weeks after introduction of *R. donacis* crawlers. For the 11-week scale density counts, low = 0–10, medium = 11–30, and high ≥ 30. * Designations follow Saltonstall [41], Saltonstall and Hauber [48].

Species/Type	Lineage/Haplotype *	# of Plants Tested	# Infested 11 Weeks	Scale Density 11 Weeks	# Infested 19 Weeks	Avg Adult Scales/Plant 19 Weeks
*Phragmites australis*	Asian/P	3	2	Medium to High	0	0
*P. australis* subsp. *berlandieri*	Gulf Coast/I	3	2	Low	0	0
*P. australis*	native X introduced hybrid	5	4	Low to Medium	3	1
*P. australis*	Introduced/M	11	10	Low to High	1	12
*P. australis* subsp. *americanus*	Native/B, E, H, S	22	18	Low to High	10	5.2
*Arundo donax*		2	2	Medium to high	2	104.5
*A. formosana*		2	0	None	2	6.5

**Table 3:** Host Range Trials of R. donacis of known native range origin (Rivesaltes, France), focusing on haplotypes of Phragmites australis and Arundo donax. Plants were dissected for development of adult female scales after 6 months (26 weeks). Adult females were isolated from plants and crawler production was summed over 8 weeks.

Species/Type	Lineage/Haplotype	# of Plants Tested	# Infested with Adult Females after 26 Weeks	Total Adults Produced	Avg Adult Female Density per Plant ^1^	# From Which Females Produced Crawlers	Total Crawlers Produced	Avg Crawler Production per Plant ^1^
*P. australis* subsp. *americanus*	Native/B, E, H, S	6	2	6	1	1	10	1.7
*P. australis* subsp. *americanus*	Native/B, E, H, S	6	0	0	0	0	0	0
*P. australis* subsp. *berlandieri*	Gulf Coast/I	5	0	0	0	0	0	0
*P. australis*	Introduced/M	5	0	0	0	0	0	0
*Arundo donax*		8	8	221	27.6	5	1010	126.3

^1^ Averages include both zero and nonzero plants as applicable.

## Data Availability

The data presented in this study are available within the article and in Supplementary Table S1.

## Supplementary Materials

The following are available online at https://www.mdpi.com/article/10.3390/insects12070588/s1, Table S1: Estimates of evolutionary divergence among sequences.
