# Supplementary material for: Biology of an Adventive Population of the Armored Scale Rhizaspidiotus donacis, a Biological Control Agent of Arundo donax in California
Source: Insects. 2021 Jun 29;12(7):588. doi: 10.3390/insects12070588 (PMC8304375; doi:10.3390/insects12070588)
Supplement: Supplementary file 1 [file insects-12-00588-s001.zip › insects-1236773-supplementary.pdf]

Table S1. Estimates of evolutionary divergence among sequences. The number of base differences per site from between sequences are shown. This analysis involved 22 nucleotide sequences. All ambiguous positions were removed for each sequence pair (pairwise deletion option). There were a total of 782 positions in the final dataset. Evolutionary analyses were conducted in MEGA X (Kumar et al. 2018)

	Villafranqueza Sp	Vingrau, France	Las Cañas, Sp	Alcoy, Sp	La P. de C., Sp	Velez de B., Sp	La Bern., Sp	Ebro River, Sp	Onda, Spain	Vil. Catalan, Fr	Coloma, Sp	Rive saltes Fr	Do. Lucata, It	Do. Lucata, It	Mar. di Noto, It	River saltes, Fr	P. Ammos, Gr	Lefkanti, Gr	Nea Artaki, Gr	Peristeri, Gr	P. Ammos, Gr
Villafranqueza, Spain																					
Vingrau, France	0.00128																				
Las Cañas, Spain	0.00128	0.00000																			
Alcoy, Spain	0.00128	0.00000	0.00000																		
La Puebla de Cazalla, Spain	0.00128	0.00000	0.00000	0.00000																	
Velez de Benaudalla, Spain	0.00128	0.00000	0.00000	0.00000	0.00000																
La Bernadilla, Spain	0.00128	0.00000	0.00000	0.00000	0.00000	0.00000															
Ebro River, Spain	0.00255	0.00128	0.00128	0.00128	0.00128	0.00128	0.00128														
Onda, Spain	0.00255	0.00128	0.00128	0.00128	0.00128	0.00128	0.00128	0.00255													
Village Catalan, France	0.00255	0.00128	0.00128	0.00128	0.00128	0.00128	0.00128	0.00255	0.00255												
Coloma, Spain	0.00128	0.00000	0.00000	0.00000	0.00000	0.00000	0.00000	0.00128	0.00128	0.00128											
Rivesaltes, France	0.00128	0.00000	0.00000	0.00000	0.00000	0.00000	0.00000	0.00128	0.00128	0.00128	0.00000										
Donna Lucata, Italy	0.03576	0.03448	0.03448	0.03448	0.03448	0.03448	0.03448	0.03576	0.03321	0.03576	0.03448	0.03448									
Donna Lucata, Italy	0.03576	0.03448	0.03448	0.03448	0.03448	0.03448	0.03448	0.03576	0.03321	0.03576	0.03448	0.03448	0.00000								
Marina di Noto, Italy	0.03193	0.03065	0.03065	0.03065	0.03065	0.03065	0.03065	0.03193	0.02937	0.03193	0.03065	0.03065	0.01660	0.01660							
Rivesaltes, France	0.00128	0.00000	0.00000	0.00000	0.00000	0.00000	0.00000	0.00128	0.00128	0.00128	0.00000	0.00000	0.03448	0.03448	0.03065						
Pohia Ammos, Greece	0.03448	0.03321	0.03321	0.03321	0.03321	0.03321	0.03321	0.03448	0.03193	0.03448	0.03321	0.03321	0.01916	0.01916	0.00511	0.03321					
Lefkanti, Greece	0.03448	0.03321	0.03321	0.03321	0.03321	0.03321	0.03321	0.03448	0.03193	0.03448	0.03321	0.03321	0.01660	0.01660	0.00255	0.03321	0.00255				
Nea Artaki, Greece	0.03576	0.03448	0.03448	0.03448	0.03448	0.03448	0.03448	0.03576	0.03321	0.03576	0.03448	0.03448	0.01788	0.01788	0.00383	0.03448	0.00383	0.00128			
Peristeri, Greece	0.03448	0.03321	0.03321	0.03321	0.03321	0.03321	0.03321	0.03448	0.03193	0.03448	0.03321	0.03321	0.01660	0.01660	0.00511	0.03321	0.00511	0.00255	0.00383		
Pachia Ammos, Greece	0.03448	0.03321	0.03321	0.03321	0.03321	0.03321	0.03321	0.03448	0.03193	0.03448	0.03321	0.03321	0.01660	0.01660	0.00255	0.03321	0.00255	0.00000	0.00128	0.00255	
California, USA	0.01022	0.00894	0.00894	0.00894	0.00894	0.00894	0.00894	0.01022	0.01022	0.01022	0.00894	0.00894	0.03065	0.03065	0.02682	0.00894	0.02937	0.02937	0.03065	0.02937	0.02937
